# National Dispensatory

**Published:** 1879-07

**Authors:** 


*The National Dispensatory; containing the Natural History, Chemistry, Pharmacy, Actions and Uses of Medicines, including those recognised in the Pharmacopœas of the United States and Great Britain.*

To a physician of forty years' active practice, whose materia medica is comprised in a list of not more than twelve or fifteen different drugs, the volume presented seems astonishingly complete and comprehensive. The general Index includes a list of over eleven thousand different substances, which are carefully described in a beautifully and substantially bound volume of 1,558 pages. It also contains a very complete and valuable "Index of Therapeutics," which will constitute a great attraction for the work.

The authors speak of the rapid progress of modern research in the group of sciences connected with Materia Medica and Therapeutics, of the new resources thus placed at the command of the pharmaceutist and physician, as justifying this great effort to make from the advanced-stand-point of the present day a concise, complete statement of all that is of practical importance to both professions. The arrangement of the work is most admirable, its typographical execution perfect, and its whole highly creditable to authors and publishers.

